# Knockdown of *Ecdysone-Induced Protein 93F* Causes Abnormal Pupae and Adults in the Eggplant Lady Beetle

**DOI:** 10.3390/biology11111640

**Published:** 2022-11-09

**Authors:** Jian-Jian Wu, Feng Chen, Rui Yang, Chen-Hui Shen, Long-Ji Ze, Lin Jin, Guo-Qing Li

**Affiliations:** Education Ministry Key Laboratory of Integrated Management of Crop Diseases and Pests, State & Local Joint Engineering Research Center of Green Pesticide Invention and Application, Department of Entomology, College of Plant Protection, Nanjing Agricultural University, Nanjing 210095, China

**Keywords:** *Henosepilachna vigintioctopunctata*, E93, larval instar, larval character, adult structure

## Abstract

**Simple Summary:**

In several hemimetabolous, neometabolous and holometabolous insects, ecdysone-induced protein 93F (E93), a critical member among three key players (Kru ppel-homolog 1, Kr-h1; E93; Broad-complex, BrC) in the regulation of metamorphosis, exerts triple roles during post-embryonic development. It acts as a determinant for the penultimate instar juveniles to attain competence, enabling the final instars to metamorphose, a repressor of juvenile characters and a specifier of adult structures during metamorphosis. However, for *Drosophila* flies whose larval instars are fixed at three, E93 only serves as an adult specifier. In a polyphagous beetle, *Henosepilachna vigintioctopunctata*, whose larval instars are fixed to four, we performed RNA interference to determine the roles of *HvE93*. Our results suggest that *Hv*E93 has dual functions, repressing larval characters and determining adult structures. Our findings provide a missing link in the evolutionary process in terms of the roles of E93.

**Abstract:**

Ecdysone-induced protein 93F (E93) plays triple roles during post-embryonic development in insects whose juvenile instars are more than four. However, it only acts as a specifier of adult structures in *Drosophila* flies whose larval instars are fixed at three. In this study, we determined the functions of E93 in the eggplant lady beetle (*Henosepilachna vigintioctopunctata*), which has four larval instars. We uncovered that *E93* was abundantly expressed at the prepupal and pupal stages. A precocious inhibition of the juvenile hormone signal by RNA interference (RNAi) of *HvKr-h1* or *HvHairy*, two vital downstream developmental effectors, at the penultimate instar larval stage increased the expression of *E93*, Conversely, ingestion of JH by the third-instar larvae stimulated the expression of *HvKr-h1* but repressed the transcription of either *HvE93X1* or *HvE93X2.* However, disturbance of the JH signal neither drove premature metamorphosis nor caused supernumerary instars. In contrast, depletion of *E93* at the third- and fourth-instar larval and prepupal stages severely impaired pupation and caused a larval-pupal mixed phenotype: pupal spines and larval scoli were simultaneously presented on the cuticle. RNAi of *E93* at the pupal stage affected adult eclosion. When the beetles had suffered from a ds*E93* injection at the fourth-instar larval and pupal stages, a few resultant adults emerged, with separated elytra, abnormally folded hindwings, a small body size and short appendages. Taken together, our results suggest the larval instars are fixed in *H. vigintioctopunctata*; E93 serves as a repressor of larval characters and a specifier of adult structures during the larval–pupal–adult transition.

## 1. Introduction

The morphological transition is of great importance in insects during post-embryonic development. Holometabolans can be separated into three successive stages: the juvenile (larval) period, the metamorphic (pupal) stage, and the reproductive adult phase. Neometabolans undergo feeding larvae, non-feeding larvae with external wing primordial (propupal and pupal stages) and adults. Hemimetabolans move from immature nymphs to adults [1,2,3]. The identity of each stage and the transformation between them rely on two hormones, 20-hydroxyecdysone (20E) and juvenile hormones (JH) [4,5,6,7,8]. By regulating the expression of three key genes encoding a C_2_H_2_ zinc finger type protein Kruppel-homolog 1 (Kr-h1), a helix-turn-helix transcription factor Ecdysone inducible protein 93F (E93), and a bric-a-brac-tramtrack-broad member Broad-Complex (BrC) [5,8], 20E and JH exert this precise developmental control. The three genes are also intimately connected to each other through a series of regulatory interactions that ensure their sequential expression to specify life stage identity, and thus are defined as the Metamorphic Gene Network (MGN) [5,9].

Among the MGN, Kr-h1 is responsible for the repression of metamorphosis during the pre-ultimate immature stages [7] by regulating the expression of both E93 and BrC [9]. Hairy, a basic helix-turn-helix transcription factor, acts synergistically with Kr-h1 to mediate JH action [10,11]. E93 plays triple roles during post-embryonic development [5]. Firstly, in the penultimate instar larvae/nymphs of a number of hemimetabolans, neometabolans and holometabolans whose juvenile instars are more than four, the presence of E93 at a low level is critical for the attainment of competence, which enables the final instars to metamorphose [4,12]. The maintenance of low E93 levels during the immature stage is based on the direct repressive action of JH through its main developmental effector, Kr-h1 [9,13,14,15,16,17,18], which is directly induced by the JH-activated receptor Methoprene-tolerant (Met)/Taiman heterodimer [19,20,21,22,23]. At the penultimate instar stage in *Tribolium castaneum*, for instance, depletion of *E93* perpetuates its juvenile identity by inducing the repetition of larval moults [13]. In immatures of the hemimetabolous *Blattella germanica*, *Schistocerca gregaria*, *Locusta migratoria*, *Gryllus bimaculatus*, *Cimex lectularius* and *Nilaparvata lugens*, depletion of *E93* disallowed the nymphal-to-adult transformation, causing supernumerary juvenile instars [15,24,25,26,27,28].

Secondly, the metamorphic transformation is involved in the presence of E93 [5] in some hemimetabolous [12,26,28], neometabolous [1,2] and holometabolous [13,29,30] insects. In the late stage of the last instar, the expression of *Kr-h1* is declined. This allows the upregulation of *E93* by the 20E-triggered Ecdysone receptor (EcR)/Ultraspiracle (USP) heterodimer [13]. The upregulated E93 then represses earlier programs that specify juvenile characters and allows youth–adult transition in several insects [13,29,30]. In numerous holometabolans, E93 facilitates the transition of young to mature organs by regulating the expression of genes involved in apoptosis and autophagy [31]. Moreover, E93 stimulates the expression of *BrC* in the prepupa. Subsequently, BrC determines the pupal stage [32]. In hemimetabolans such as *L. migratoria*, RNAi of *E93* prevents the destruction of old nymphal cuticles [28].

Thirdly, E93 acts as an adult specifier [5]. Very high levels of E93 have been reported in the pupal stage in holometabolous insects and in the propupal and pupal stages in neometabolous Thysanopterans [2,9,13]. Knockdown of *E93* in *T. castaneum* pupae inhibits adult transition and brings about an extra second pupae, clearly signifying that E93 serves as an adult specifier [26].

In *Drosophila melanogaster*, the maintenance of larval identity and the repression of larval characters do not depend on E93. A loss of E93 at the penultimate and final instars does not affect larval–pupal–adult transformation by the end of the third larval instar [26,33]. Removal of E93 exerts little effect on either the expression of death genes or the destruction of larval salivary glands [4], even though ectopic expression of E93 is enough to elicit cell death in imaginal discs, Malpighian tubules, embryonic epithelial cells, and larval fat bodies [34,35,36,37]. E93 has ever been proposed to contribute to the stage-specific initiation of key death inducer genes [34,35]; however, the mutants used for these studies carried lesions in a neighboring locus encoding isocitrate dehydrogenase 3b, an enzyme in the tricarboxylic acid cycle that is vital for the cell death of salivary glands [38]. Consistently, mutants of either Kr-h1 or the JH receptor do not undergo premature metamorphosis [39,40,41]. Therefore, larval instars are fixed at three in *D. melanogaster*, E93 does not serve as a metamorphosis-activating factor [5]. Conversely, the adult specifier role of E93 is conserved in *D. melanogaster* [26]; *E93* mutants die as pharate adults [33].

The eggplant lady beetle (*Henosepilachna vigintioctopunctata*), a defoliating pest attacking Solanaceae and Cucurbitaceae crops in many Asian countries [42], only has four instars. Disruption of the 20E signal fails to bring about supernumerary instars or second pupae in *H. vigintioctopunctata* [43,44]. We accordingly hypothesized that the larval instars in the beetle were fixed. In this study, we sought to test the hypothesis in *H. vigintioctopunctata*. We knocked down the target gene and observed the negative phenotypes. Our findings support the hypothesis and ascertain the dual functions of E93 during larval–pupal–adult transformation: as a repressor of larval characters and as a specifier of adult structures.

## 2. Materials and Methods

### 2.1. Insect

The *H. vigintioctopunctata* beetles were reared using fresh potato foliage at the vegetative growth or young tuber stages [43,44]. At this feeding protocol, the ladybirds progressed through four distinct larval instars (3, 2, 2 and 3 days, respectively), prepupal (2 days), pupal (4 days) and adult stages.

### 2.2. Molecular Cloning and Bioinformatic Analysis

*E93*, *Kr-h1* and *Hairy* genes were mined from the transcriptome database of *H. vigintioctopunctata* [42]. The correctness of the sequence of *E93* was validated by polymerase chain reaction (PCR) using primers in Figure S1 and Table S1. The corrected cDNA sequences were uploaded to GenBank (accession numbers: *HvE93X1*, OM001097; *HvE93X2*, OM001098).

The total RNA was isolated using TRIzol reagent (Invitrogen, New York, NY, USA) in accordance with the manufacturer’s protocols. The quality and quantification of total RNA were assessed by the NanoDrop 2000 spectrophotometer (Thermo Fisher Scientific, New York, NY, USA). The RNA integrity was examined via 1% agarose gel electrophoresis. The cDNA was produced using a PrimeScript^TM^ RT reagent Kit with a gDNA eraser (TaKaRa Biotechnology Co., Ltd., Dalian, China), incubated at 37 °C for 15 min and then at 85 °C for 5 s.

The protein sequences of E93 from other species were downloaded from the NCBI. The domains of *Hv*E93s were identified by NCBI Conserved Domain Search (https://www.ncbi.nlm.nih.gov/Structure/cdd/wrpsb.cgi accessed on 12 June 2021). The HTH_psq domains of the *Hv*E93 isoforms were compared with those derived from *L. decemlineata*, *S. gregaria* and *B. germanica* by GENEDOC software. Phylogenetic analysis was achieved by MEGA 6.0 and the neighbor-joining method with 1000 bootstrap replications.

### 2.3. Synthesis of dsRNA Molecules

Two cDNA fragments targeting *HvKr-h1* (ds*Kr-h1*-1 and ds*Kr-h1*-2), *HvHairy* (ds*Hairy*-1 and ds*Hairy*-2), or the common sequences of both *HvE93* isoforms (ds*E93*-1 and ds*E93*-2) were chosen (Table S1). A cDNA sequence derived from the enhanced green fluorescent protein (ds*egfp*) was used as a control (Table S1). These targeted regions were further BLAST (BLASTN) searched against the *H. vigintioctopunctata* transcriptome to identify any possible off-target sequences that had an identical match of 20 bp or more. These cDNA fragments were respectively amplified by PCR using specific primers (Table S1) conjugated with the T7 RNA polymerase promoter. The dsRNA was synthesized using the MEGAscript T7 High Yield Transcription Kit (Ambion, Austin, TX, USA) according to the manufacturer’s instructions. The synthesized dsRNA was determined by agarose gel electrophoresis and the Nanodrop 1000 spectrophotometer.

### 2.4. Introduction of dsRNA

A described method was used to inject dsRNA [45,46]. Briefly, 500 ng of dsRNA (0.1 μL) was injected into the body cavity of the newly ecdysed fourth instar larvae, the prepupae and the pupae, and 300 ng of dsRNA was introduced into the newly molted third instar larvae. Negative controls were injected with the same volume of ds*egfp* solution.

Seven biologically independent experiments were performed using the newly molted fourth or third instar larvae from different generations or the newly formed prepupae and pupae. The first and second bioassays were designed to test whether the JH signal regulates the expression of *HvE93X1* and *HvE93X2* in the third instar larvae. The first experiment had five treatments: (1) ds*egfp*, (2) ds*Kr-h1*-1, (3) ds*Kr-h1*-2, (4) ds*Hairy*-1 and (5) ds*Hairy*-2. The second bioassay was performed by confining the larvae in petri dishes containing potato foliage immersed in a JH solution at a concentration of 0, 50, 100 or 200 ng/mL. The third to sixth bioassays were planned to measure the RNAi effects of both *HvE93* isoforms in third-, fourth-instar larvae, prepupae and pupae, and had three treatments: (1) ds*egfp*, (2) ds*E93*-1 and (3) ds*E93*-2. The seventh bioassay was rescuing experiments (100 ng/mL 20E) in the fourth-instar larvae, and had twelve treatments: (1) ds*egfp*, (2) ds*E93*-1, (3) ds*E93*-2, (4) ds*egfp* + ds*Kr-h1*-1, (5) ds*egfp* + ds*Kr-h1*-2, (6) ds*egfp* + 20E, (7) ds*E93*-1 + 20E, (8) ds*E93*-2 + 20E, (9) ds*E93*-1 + ds*Kr-h1*-1, (10) ds*E93*-2 + ds*Kr-h1*-1 and (11) ds*E93*-1 + ds*Kr-h1*-2, (12) ds*E93*-2 + ds*Kr-h1*-2. Each bioassay was repeated nine times; a replicate consisted of 10 injected individuals. To test RNAi efficacy, three repeats were collected 24 h, 36 h or 72 h after injection for qRT-PCR. Three replicates were used to measure the weight and to observe the phenotypes during a 3-week trial period. Another three repeats were collected and dissected to observe and image the phenotypes under a microscope.

### 2.5. Real-Time Quantitative PCR (qRT-PCR)

To test temporal expression, beetle specimens, including 3-day-old eggs and the neonates through adults, were picked up. To analyze the tissue expression patterns, the 2-day-old fourth instar larvae were dissected, and the foregut, midgut, hindgut, Malpighian tubules, epidermis and fat body were collected. Moreover, treated larval samples were collected 3 days after dsRNA injection. Each specimen consisted of 20–30 individuals and was repeated three times. According to the manufacturer’s instructions, the RNA was extracted and purified using the SV Total RNA Isolation System Kit (Promega) and DNase I, respectively. Real-time quantitative PCR was performed in technical triplicate, using two internal control genes (*HvRPS18* and *HvRPL13*) (Table S1) [47]. The generation of specific PCR products was confirmed by gel electrophoresis [48]. Data were given as the 2^−ΔΔCT^ or 2^−ΔCT^ values.

### 2.6. Data Analysis

Statistical analyses were performed using SPSS for Windows (Chicago, IL, USA). Following the assurance of a normal distribution of data, one-way analysis of variance (ANOVA) with the Tukey–Kramer post hoc test was used for multiple comparisons. Some data were compared using a *t*-test. Values (Mean ± SE) of *p* < 0.05 were regarded as significant. Since the differences in RNAi efficacy and defective phenotypes between ds*E93*-1 and ds*E93*-2, ds*Kr-h1*-1 and ds*Kr-h1*-2, ds*Hairy*-1 and ds*Hairy*-2 were not significant, the data for the genes were combined.

## 3. Results

### 3.1. Identification of E93 Isoforms in H. vigintioctopunctata

Two full-length *HvE93* cDNAs (*HvE93X1* and *HvE93X2*) were identified (Figure S1). They, respectively, contained 2976 bp and 2988 bp of open reading frames. The predicted proteins possess 991 and 995 amino acid residues, respectively (Figure S2). Both predicted E93 isoforms have two helix-turn-helix (HTH) psq-type motifs (Figure S2) that are considered to form a HTH DNA binding domain [49].

A phylogenetic tree was constructed using E93 proteins from representative insect species (Figure S3). The E93 proteins formed hemimetabolan and holometabolan clades. However, the position of *Drosophila* E93 was not ascertained due to low bootstrap support. In the Coleoptera clade, two E93 isoforms from *H. vigintioctopunctata* were first grouped with 100% bootstrap support, forming a subclade; this subclade was then joined with that from *Leptinotarsa decemlineata*, with a bootstrap support of 99% (Figure S3).

### 3.2. The Expression Profiles of HvE93, HvKr-h1 and HvHairy

**Figure 1:**
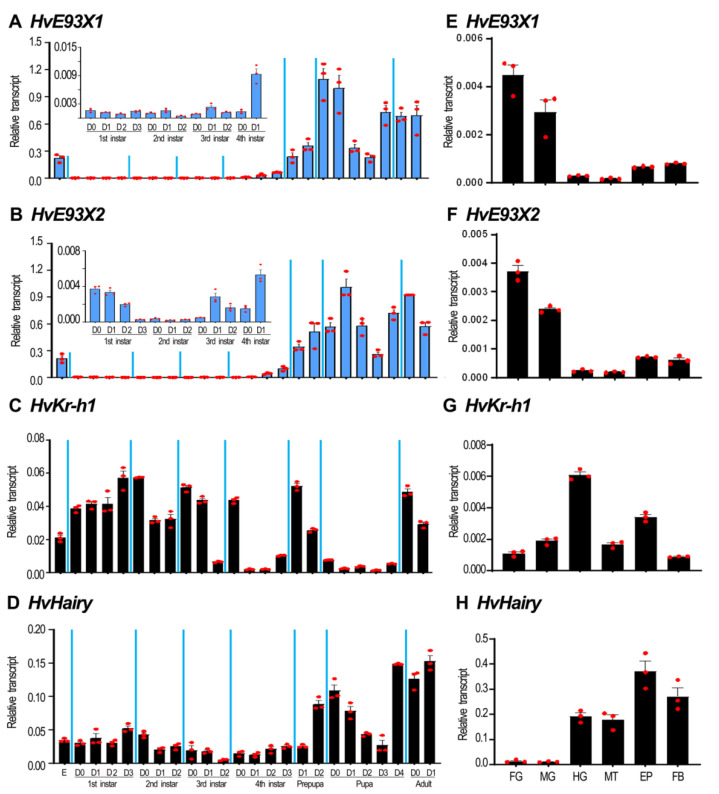
**Temporal (A–D) and tissue (E–H) transcription patterns of *HvE93X1*, *HvE93X2*, *HvKr-h1* and *HvHairy* in *Henosepilachna vigintioctopunctata*.** RNA templates for temporal analysis were derived from eggs (3-day-old), the larvae from the first through the fourth instars, prepupae, pupae and adults (D0 indicated newly ecdysed larvae or pupae, or newly emerged adults), or from the foregut (FG), midgut (MG), hindgut (HG), Malpighian tubules (MT), epidermis (EP) and fat body (FB) of the 2-day-old larvae for tissue-biased transcription analysis. The columns (2^−ΔCT^ values) represent averages, with vertical lines indicating SE. Inserts in panel A and panel B are amplified expression levels in 0-day-old neonates through 1-day-old fourth-instar larvae.

Given that the expression of *E93* was regulated by the JH signal [9,13,14,15,16,17,18], the expression patterns of *HvE93X1*, *HvE93X2*, *HvKr-h1* and *HvHairy* were measured during different developmental stages (Figure 1). Their mRNAs were easily detected from embryo (egg) to adult (Figure 1A–D). The high levels of the two *HvE93* isoforms were found in the embryonic, prepupal, pupal and adult stages (Figure 1A,B). In contrast, the abundant mRNA levels of *HvKr-h1* were measured in the first and second instar larvae, the young third and fourth instar larvae, the prepupae and the adults (Figure 1C). For *HvHairy*, its transcription levels were high in the old prepupae, pupae and adults (Figure 1D).

In the 2-day-old, fourth-instar larvae, *HvE93X1*, *HvE93X2*, *HvKr-h1* and *HvHair* were actively transcribed in the guts, Malpighian tubules, epidermis and fat body. Either *HvE93X1* or *HvE93X2* was highly expressed in the foregut and midgut, moderately transcribed in the epidermis and fat body, and lowly expressed in the hindgut and Malpighian tubules (Figure 1E,F). In contrast, the highest and second highest-levels of *HvKr-h1* were found in the hindgut and epidermis, followed by those in the midgut and Malpighian tubules, and the lowest levels were measured in the fat body and foregut (Figure 1G). For *HvHairy*, it was abundantly expressed in the epidermis and fat body, temperately transcribed in the hindgut and Malpighian tubules, and poorly expressed in the foregut and midgut (Figure 1H).

### 3.3. JH signal Inhibits the Expression of HvE93

**Figure 2:**
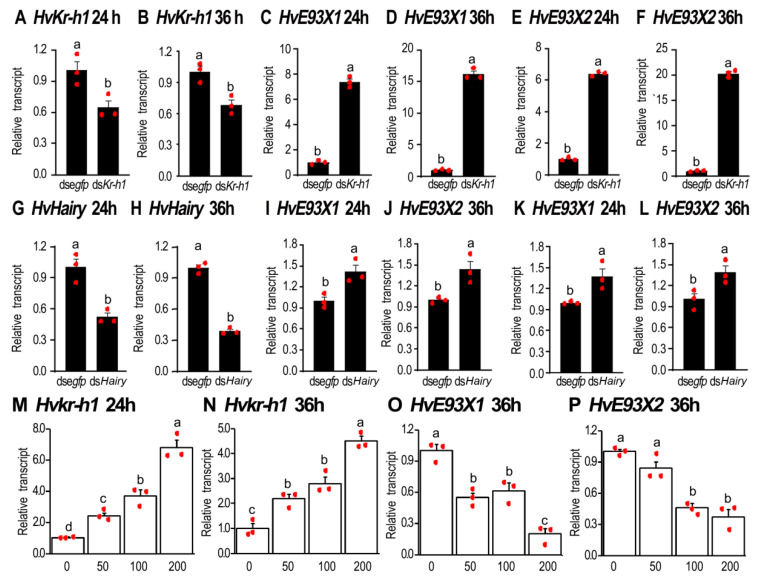
**Disruption of the JH signal affects the expression of *HvE93X1* and *HvE93X2* in *Henosepilachna vigintioctopunctata*.** Around 300 ng of ds*Kr-h1*, ds*Hairy* or ds*egfp* (0.1 μL) was injected into the newly-molted third instar larvae (**A**–**L**). Moreover, the ladybirds were confined in petri dishes containing potato foliage immersed with a JH solution at a concentration of 0, 50, 100 or 200 ng/mL (**M**–**P**). Twenty-four and thirty-six hours after injection, the expression levels (2^−ΔΔCT^ values) of the target gene (either *HvKr-h1* or *HvHairy*), *HvE93X1* and *HvE93X2* were estimated. Different letters above the columns (means± SE) indicate a significant difference at *p* < 0.05 using an independent sample *t*-test (**A**–**L**) or an analysis of variance with the Tukey–Kramer test (**M**–**P**).

In order to test whether JH signaling regulates the expression of *HvE93X1* and *HvE93X2*, either *HvKr-h1* or *HvHairy* was depleted by injection of the corresponding dsRNA at the young third instar larval stage (Figure 2A,B,G,H). Knockdown of *HvKr-h1* highly enhanced the expression of either *HvE93X1* or *HvE93X2* (Figure 2C–F). Similarly, RNAi of *HvHairy* mildly increased the transcription of both isoforms (Figure 2I–L). Conversely, ingestion of JH by the third-instar larvae stimulated the expression of *HvKr-h1* but repressed the transcription of either *HvE93X1* or *HvE93X2* (Figure 2M–P).

However, any negative effects on the metamorphosis were observed in *HvKr-h1* or *HvHairy* RNAi larvae or JH-ingested ladybirds.

### 3.4. HvE93 Depletion in the Third Instar Larva Affects Larval-Pupal Transition

**Figure 3:**
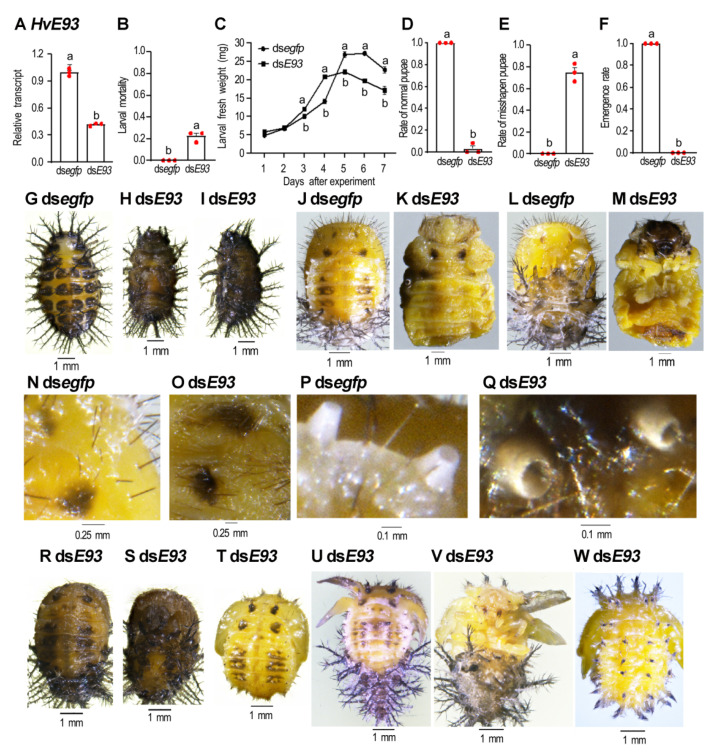
**Knockdown of both *HvE93* isoforms in the penultimate instar larvae impair larval-pupal-adult transition in *Henosepilachna vigintioctopunctata*.** Around 300 ng of ds*E93* or ds*egfp* (0.1 μL) was injected into the newly molted third instar larvae. The ladybirds were subsequently confined in petri dishes containing potato foliage. Three days after injection, the expression level (2^−ΔΔCT^ values) of *HvE93* was measured (**A**). The larval mortality (**B**), rates of normal and deformed pupae (**D**,**E**), and emergence rate (**F**) were recorded during a 3-week trial period. The resultant larvae were weighed 1 through 7 days after treatment (**C**). Statistical significances (*p* value < 0.05, showing by different letters above the columns) between treatment and control (means ± SE) were calculated using an independent sample *t*-test. Dorsal (**G**,**H**) and lateral (**I**) views of arrested larvae are shown. After the removal of the larval exuviae, the dorsal (**K**) and ventral (**M**) views of the resultant prepupae are shown, compared with the control pupae (**J**,**L**). The black markings and pupal spines (**O** vs. **N**), and two abdomen spiracles (**Q** vs. **P**) of treated and control beetles are further amplified. The dorsal (**R**,**T**,**U**,**W**) and ventral (**S**,**V**) views of the resultant *E93* RNAi pupae showed the defective phenotypes.

Three days after ds*E93* injection, the *HvE93* mRNA level was significantly reduced in the treated beetles, compared with that in the control larvae (Figure 3A).

The control beetles normally underwent the third-fourth instar molting and grew up (Figure 3B,C,G). Similarly, all beetles previously exposed to ds*E93* became fourth-instar larvae, with smaller fresh weight (Figure 3C) and body size (Figure 3H,I vs. Figure 3G). A total of 22% *HvE93* RNAi fourth-instar larvae arrested developing at the prepupal stage; they darkened, dried and finally died (Figure 3B,H,I). After removal of the larval exuviae, the pupal sharps were observed for these *HvE93* RNAi beetles (Figure 3K,M vs. Figure 3J,L). The characteristic structures, such as black markings and pupal spines (Figure 3O vs. Figure 3N), the pupal spiracles (Figure 3Q vs. Figure 3P) were well formed on the cuticles of these *HvE93* RNAi beetles.

After shedding the fourth-instar larval exuviae, 76% of the ds*E93*-treated beetles directly formed misshapen pupae (Figure 3E). Less than 2% formed pupae (Figure 3D), with smaller body sizes (Figure 3R,S vs. Figure 3J). These pupae did not emerge as adults.

Around 76% of these misshapen *HvE93* RNAi pupae had normal (Figure 3T) or expanded and stretched (Figure 3U–W) wing discs. Moreover, the cuticular surface of a control fourth-instar larva was covered with ten rows of dark branched spines (scolus) located on black epidermis mastoids (struma) (Figure 3G), whereas the control pupae were covered with fine spines (Figure 3J). In the misshapen *HvE93* RNAi pupae, in contrast, both pupal spines and the larval scoli were seen (Figure 3T–W). No misshapen *HvE93* RNAi pupae emerged as adults (Figure 3F).

### 3.5. HvE93 Knockdown in the Fourth-Instar Larvae Impairs Larval–Pupal–Adult Transition

**Figure 4:**
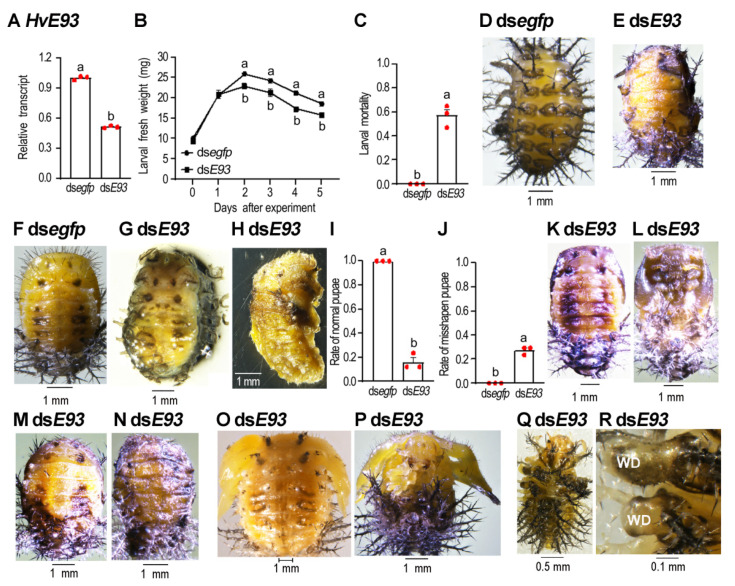
**Depletion of both *HvE93* isoforms in the final instar larvae arrests the development of *Henosepilachna vigintioctopunctata* larvae.** Around 500 ng of ds*E93* or ds*egfp* (0.1 μL) was injected into the newly molted fourth-instar larvae. The ladybirds were subsequently confined in petri dishes containing potato foliage. Three days after injection, the expression level (2^−ΔΔCT^ values) of *HvE93* was measured (**A**). The resultant larvae were weighed 1 through 5 days after treatment (**B**). The larval mortality (**C**), rate of normal pupae (**I**) and rate of misshapen pupae (**J**) were recorded during a 3-week trial period. Different letters above the data (means± SE) show a significant difference at *p* < 0.05 using an independent sample *t*-test. The resultant larvae arrest development at the prepupal stage (**E** vs. **D**). After removal of the larval exuviae, the dorsal (**G**) and lateral (**H**) views of the resultant prepupae are shown, compared with the control pupa (**F**). The defective phenotypes of the treated pupae are shown (**M**–**Q**), compared with the control (**F**). The thorax of the treated pupae in (**Q**) are amplified (**R**) to show stretched fore- and hindwing discs (WD).

Three days after injection of 500 ng ds*E93* into the body cavity of newly molted four-instar larvae, the *HvE93* mRNA level was significantly lowered in the treated beetles, compared to controls (Figure 4A).

The *HvE93* RNAi fourth-instar larvae grew more slowly and obtained a lighter fresh weight (Figure 4B) and a smaller body size (Figure 4E vs. Figure 4D) than controls. Around 59% of the *HvE93* knockdown larvae did not shed their old larval cuticle (Figure 4C,E). After removal of the larval exuviae, the pupal sharps were seen for these *HvE93* RNAi beetles (Figure 4G,H vs. Figure 4F).

The remaining 41% of the *HvE93* RNAi beetles partially or completely shed the fourth-instar larval exuviae. About 18% of the *HvE93* hypomorphs normally pupated (Figure 4I), with normal wing discs (Figure 4K–N vs. Figure 4F). Among the 18% of the *HvE93* hypomorphs, only one fifth of them completely shed their old exuviae and formed normal pupae with darkened pupal cuticles (Figure 4K,L vs. Figure 4F). The remaining four fifths of the *HvE93* hypomorphs formed miniature pupae, partially wrapped in their old exuviae (Figure 4M,N). They finally dried up and died.

Around 23% of the *HvE93* knockdown beetles became misshapen pupae (Figure 4J). Two defective phenotypes were observed: around half of the *HvE93* RNAi hypomorphs successfully shed the old exuviae, bearing expanded wing discs. Both pupal spines and the larval scoli were seen (Figure 4O,P). Another half of the *HvE93* RNAi hypomorphs failed to shed their old exuviae. However, the wing discs were expanded and stretched outside the larval cuticle (Figure 4Q,R).

**Figure 5:**
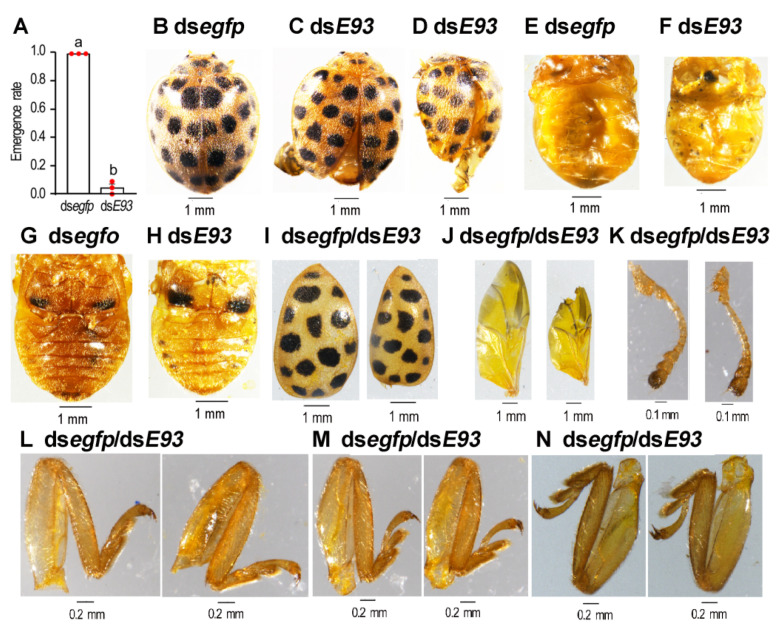
**RNAi of both *HvE93* isoforms in the final instar larvae affects adult emergence in *Henosepilachna vigintioctopunctata* larvae.** The emergence rates of the treated and control ladybirds were recorded during a 3-week trial period (**A**). Statistical significances (*p* value < 0.05, showing by different letters above the columns) between treatment and control (means ± SE) were calculated using an independent sample *t*-test. The defective phenotypes of the treated adults (**C**,**D**) are shown, compared with the control (**B**). The sizes of the abdomen (**F**,**H** vs. **E**,**G**), the elytrum (**I**), the hindwing (**J**), the antennae (**K**), the foreleg (**L**), the midleg (**M**) and the hindleg (**N**) of the treated adults were compared with the corresponding organs from controls (right vs. left panel).

Less than 4% of the *HvE93* knockdown beetles successfully emerged as adults (Figure 5A). These adults had separated elytra and unfolded hindwings (Figure 5C,D vs. Figure 5B). The sizes of the abdomen (Figure 5F,H vs. Figure 5E,G), the elytrum (Figure 5I), the hindwing (Figure 5J), the antennae (Figure 5K), the foreleg (Figure 5L), the midleg (Figure 5M) and the hindleg (Figure 5N) were smaller than the corresponding organs from the control adults, although the differences in the legs were not so obvious (Figure 5L–N). The *HvE93* knockdown adults neither moved nor fed on foliage, and all of them died within one week after emergence. 

### 3.6. HvE93 RNAi in the Prepupae Mimicks the Impairment of Metamorphosis

**Figure 6:**
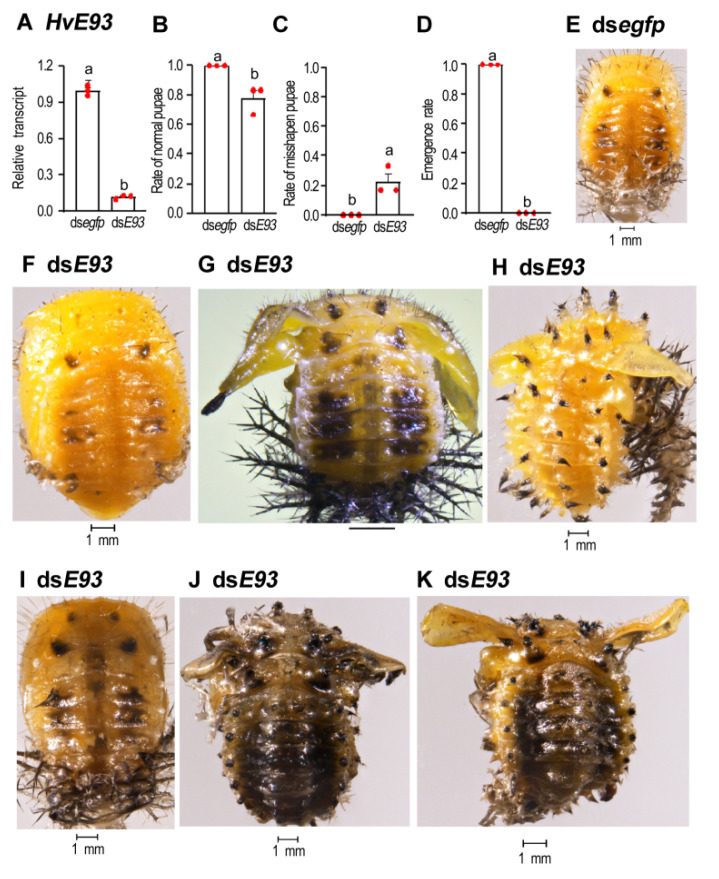
**Knockdown of both *HvE93* isoforms in the prepupae influences pupation and adult emergence in *Henosepilachna vigintioctopunctata*.** Around 500 ng of ds*E93* or ds*egfp* (0.1 μL) was injected into the newly formed prepupae. One day after injection, the expression level (2^−ΔΔCT^ value) of *HvE93* was measured (**A**). Rates of normal and deformed pupae (**B**,**C**) and emergence rate (**D**) were recorded during a 3-week trial period. Different letters above the columns (means± SE) show a significant difference at *p* < 0.05 using an independent sample *t*-test. Two defective phenotypes were noted: normal pupae (**F**) and wing-stretched pupae with both pupal spines and larval scoli on the cuticle (**G**,**H**), compared to the control pupa (**E**). The treated pupae become darkened and dried (**I**–**K**). No treated pupae emerge as adults (**D**).

RNAi of *HvE93* (Figure 6A) in the prepupae almost mimicked the defective phenotypes in the *HvE93* depletion fourth-instar larvae (Figure 5). Around 80% of the *HvE93* knockdown pupae appeared normal (Figure 6B,F vs. Figure 6E). The remaining 20% of the *HvE93* hypomorphs formed misshapen pupae (Figure 6C,G,H). They possessed expanded and stretched wings. Moreover, both pupal spines and larval scoli were observed on the cuticle (Figure 6G,H).

All the *HvE93* depletion pupae darkened, dried up and died (Figure 6I–K). No adults emerged (Figure 6D).

### 3.7. Depletion of HvE93 in the Pupae

**Figure 7:**
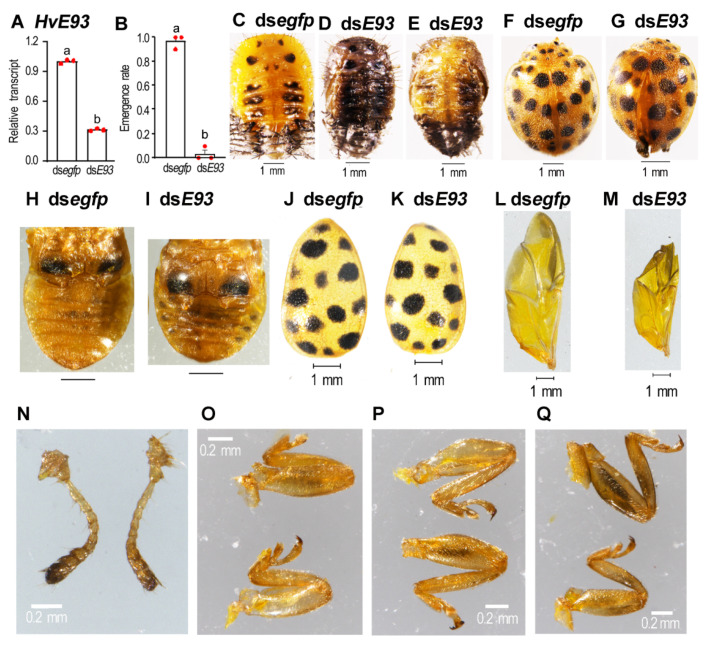
**Depletion of both *HvE93* isoforms in the pupae causes failure of adult emergence in *Henosepilachna vigintioctopunctata*.** Around 500 ng of ds*E93* or ds*egfp* (0.1 μL) was injected into the newly formed pupae. Three days after injection, the expression level (2^−ΔΔCT^ value) of *HvE93* was measured (**A**). The emergence rate was recorded during a 3-week trial period (**B**). Statistical significances (*p* value < 0.05, showing by different letters above the columns) between treatment and control (means± SE) were calculated using an independent sample *t*-test. Most treated pupae became darkened and dried (**D**,**E**), compared to normal pupae (**C**). A small portion of treated pupae emerge as adults (**B**), with deformed wings (**G** vs. **F**). The sizes of the abdomen (**I** vs. **H**), the elytrum (**K** vs. **J**), the hindwing (**M** vs. **L**), the antennae (**N**, **left** vs. **right**), the foreleg (**O**, **below** vs. **above**), the midleg (**P**, **below** vs. **above**) and the hindleg (**Q**, **below** vs. **above**) of the treated adults were compared with the corresponding organs from controls.

Three days after injection of 500 ng ds*E93* into the abdomen of newly formed pupae, *HvE93* mRNA level was significantly lowered in treated beetles, compared with control (Figure 7A).

Around 96% of the *HvE93* RNAi pupae gradually became darkened and wrinkled (Figure 7D,E vs. Figure 7C), they finally died. Only 4% of the *HvE93* knockdown pupae emerged as adults (Figure 7B). The resultant adults had separated elytra and unfolded hindwings (Figure 7G vs. Figure 7F). The *HvE93* RNAi adults had smaller abdomens (Figure 7I vs. Figure 7G), elytrums (Figure 7K vs. Figure 7J), hindwings (Figure 7M vs. Figure 7L), forelegs (Figure 7O, below vs. above ones), midlegs (Figure 7P, below vs. above ones) and hindlegs (Figure 7Q, below vs. above ones) than control beetles, although the differences in the antennae were not obvious (Figure 7N, right vs. left ones). They hardly moved or fed on foliage; all of them eventually died within a week.

### 3.8. RNAi of HvE93 Disrupts 20E and JH Signals

**Figure 8:**
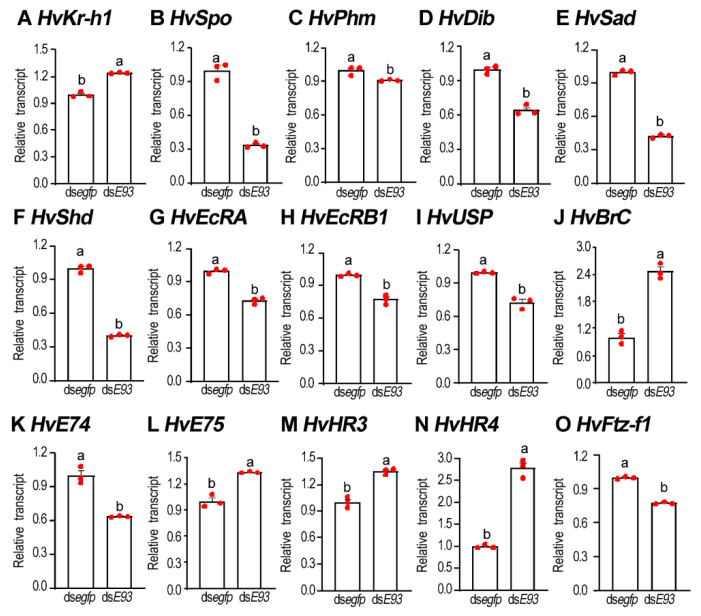
**The expression levels of JH and 20E signal genes in the *HvE93* fourth-instar larvae in *Henosepilachna vigintioctopunctata*.** Around 500 ng of ds*E93* or ds*egfp* (0.1 μL) was injected into the newly molted fourth-instar larvae. Three days after injection, the expression levels (2^−ΔΔCT^ values) of *HvKr-h1*, five Halloween genes (*HvSpo*, *HvPhm*, *HvDib*, *HvSad* and *HvShd*), three 20E receptor genes (*HvEcRA*, *HvEcRB1* and *HvUSP*) and six 20E response genes (*HvBrC*, *HvE74*, *HvE75*, *HvHR3*, *HvHR4* and *HvFtz-f1*) were determined. Statistical significances (*p* value < 0.05, showing by different letters above the columns) between treatment and control (means ± SE) were calculated using an independent sample *t*-test.

The mRNA levels of *HvKr-h1* were measured in the resultant prepupae whose fourth-instar larvae had suffered from ds*E93* injection. As expected, the level was significantly upregulated in treated beetles, compared with control (Figure 8A).

The transcription profiles of five Halloween genes, *Spook* (*HvSpo*), *Phantom* (*HvPhm*), *Disembodied* (*HvDib*), *Shadow* (*HvSad*) and *Shade* (*HvShd*), which encode cytochrome P450 enzymes involved in ecdysteroid biosynthesis, were compared between ds*E93*- and ds*egfp*-injected fourth-instar larvae 3 days after treatment (Figure 8B–F). Relative mRNA levels of the five Halloween genes in the ds*E93*-injected beetles were significantly lower, compared with those in the control prepupae (Figure 8B–F).

Moreover, we have also analyzed the transcript levels of the ecdysone receptor complex genes *HvEcRA*, *HvEcRB1* and *HvUSP* in both *HvE93* knockdown and ds*egfp* control animals. When compared with control (ds*egfp*) ladybirds, significant decreases in transcript levels of *HvEcRA*, *HvEcRB1* and *HvUSP* were observed in the ds*E93*-injected prepupae (Figure 8G–I).

Furthermore, the expression levels of six 20E response genes were determined. Compared with the control beetles, the transcription levels of *HvBrC*, *HvE75*, *HvHR3* and *HvHR4* were significantly upregulated, whereas the mRNA levels of *HvE74* and *HvFtz-f1* were significantly downregulated in the *HvE93* RNAi prepupae (Figure 8J–O).

### 3.9. Rescuing Experiments with 20E or a Combination of dsE93 + dsKr-h1

**Figure 9:**
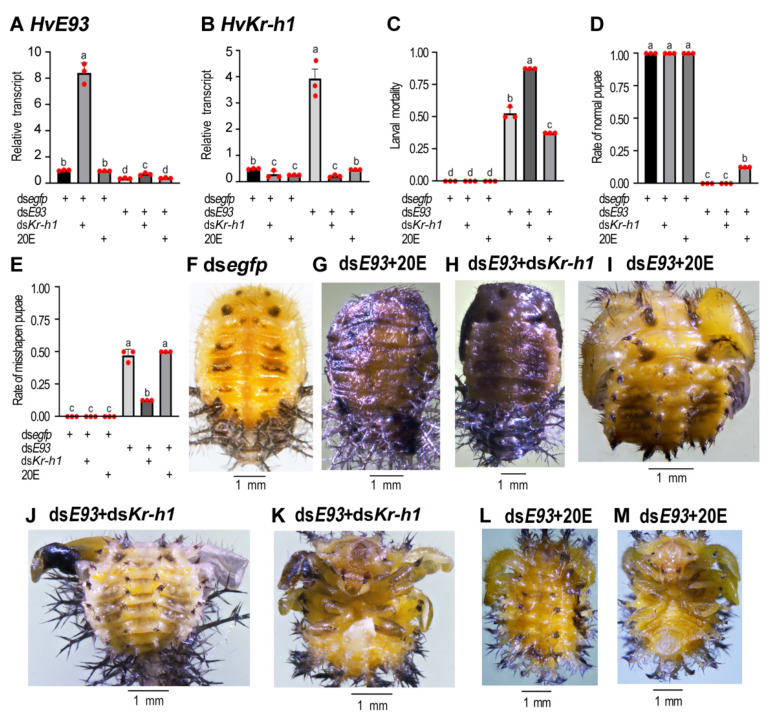
**Combined effects of ds*E93* and 20E, or ds*E93* and ds*Kr-h1*, on the development.** The newly molted four-instar larvae were treated with ds*egfp*, ds*egfp* + ds*Kr-h1*, ds*egfp* + 20E, ds*E93*, ds*E93* + ds*Kr-h1* or ds*E93* + 20E. Three days after treatment, transcript levels (2^−ΔΔCT^ values) of *HvE93* and *HvKr-h1* were determined. The rates of arrested larvae, normal and deformed pupae were recorded during a 3-week trial period (**C**–**E**). Statistical significances (*p* value < 0.05, showing by different letters above the columns) between treatment and control (means ± SE) were calculated using analysis of variance with the Tukey–Kramer test. Dorsal (**F**–**J**,**L**) and ventral (**K**,**M**) views of pupae are shown.

Given that the 20E biosynthesis pathway was repressed, whereas the expression of *HvKr-h1* was enhanced in the *HvE93* RNAi beetles, we performed rescuing experiments by dietary supplement with 20E or RNAi of both *HvKr-h1* and *HvE93* (Figure 9).

Ingestion of 20E by 3-day-old, fourth-instar larvae hardly affected the mRNA levels of *HvE93* in both ds*egfp*- and ds*E93*-injected beetles (Figure 9A). In contrast, knockdown of *HvE93* greatly increased the mRNA level of *HvKr-h1* (Figure 9B). In the resultant beetles, having received a combination of ds*E93* + ds*Kr-h1* injections, the mRNA levels of both *HvE93* and *HvKr-h1* were significantly reduced, compared with those in the control prepupae (Figure 9A,B).

Feeding of 20E by *HvE93* RNAi larvae partially alleviated the larval mortality (Figure 9C) and relieved the normal pupation (Figure 9D). However, consumption of 20E did not restore the rate of misshapen miniature pupae in the *HvE93* RNAi beetles (Figure 9E). Around one third of the small, deformed pupae became dark and dried (Figure 9G vs. Figure 9F); another two thirds possessed both pupal spines and larval scoli (Figure 9I,L), bearing expanded (Figure 9I) or stretched (Figure 9L,M) wings.

Although injection of ds*Kr-h1* alone did not cause lethality, a combination of ds*E93* + ds*Kr-h1* injection led to more larval mortality (Figure 9C) but less misshapen miniature pupae (Figure 9D,E). Similar phenotypes, such as dark and dried (Figure 9H), simultaneously presented pupal spines and larval scoli (Figure 9J), and expanded and stretched wings (Figure 9J,K) were noted in the ds*E93* + ds*Kr-h1* treated beetles.

## 4. Discussion

Temporal regulation during development ensures each stage occurs at the appropriate time, for the proper duration, and in the correct order [4]. In the present paper, we uncovered that the temporal expression pattern of *E93* (Figure 1) is comparable with the corresponding genes in pupal stages in holometabolous insect *T. castaneum* and *D. melanogaster*, and propupal and pupal stages in neometabolous Thysanopterans [2,4,9,13,26]. Moreover, RNAi of *Kr-h1* (Figure 2) and *E93* (Figure 8 and Figure 9) and ingestion of JH (Figure 2) demonstrate that the mutual regulation of *E93*, *Kr-h1* and *BrC* in *H. vigintioctopunctata* is similar to other insects [26], indicating that the role of the Metamorphic Gene Network (MGN) is conserved [5,9]. Moreover, our findings here support the pivotal roles for E93 acting as a repressor of larval characters and as a specifier of adult structures during metamorphosis.

### 4.1. The Number of Larval Instars Is Fixed in H. vigintioctopunctata

In this study, we revealed that the number of larval instars was default in *H. vigintioctopunctata*, just like that in *D. melanogaster* [5]. Our conclusion was clearly shown by the following two results. Firstly, a precocious inhibition of JH signal by RNAi of either *HvKr-h1* or *HvHairy* or an addition of JH at the penultimate instar larval stage did not affect normal metamorphosis in *H. vigintioctopunctata* (Figure 2), similar to the results in *Kr-h1*, *Met* or *Gce* mutants in *D. melanogaster* [39,40,41]. Conversely, RNAi of *Kr-h1* triggers precocious adults in hemimetabolans and induces premature pupation in holometabolans [24,25,29]. For example, depletion of *Kr-h1* in *Bombyx mori* causes precocious larval–pupal and larval–adult metamorphosis during the pre-ultimate larval stages [17,28]. In *T. castaneum*, injection of ds*Kr-h1* into the late penultimate instar larvae triggers the metamorphic transition before the attainment of threshold size [13]. Injection of ds*Kr-h1* into the final larval instar impairs ecdysis. Under the treated larval exuviae, the adults have precociously developed, bypassing the pupal stage [9].

Secondly, the *HvE93* RNAi larvae initiated a timely metamorphic transition by the end of the final larval stage (Figure 3), comparable to the results in the *E93* depleted *Drosophila* larvae [26,33]. In contrast, depletion of *TcE93* by dsRNA injection in penultimate instar (L6) larvae in *T. castaneum* gives rise to supernumerary larval instars (L8). A reinjection of the RNAi L8 larvae causes L9 (65%) or even L10 (35%) larvae [13].

The two pieces of experimental evidence in this survey indicate that the number of larval instars is fixed in *H. vigintioctopunctata*.

### 4.2. E93 Acts as a Repressor of Larval Scoli during Metamorphosis

In this survey, we unravel that pupal spines and larval scoli were simultaneously presented on the cuticle of the *E93* RNAi pupae in *H. vigintioctopunctata* (Figure 3, Figure 4, Figure 6 and Figure 9). In accordance with our results, RNAi of *E93* in *L. migratoria* prevents the destruction of old nymphal cuticles [28]. It is well known that ectopic expression of *E93* is sufficient to activate cell death in imaginal discs, Malpighian tubules, embryonic epithelial cells and larval fat body [34,35,36,37]. Knockdown of *E93* may thus repress the apoptosis and autophagy of the larval epithelial cells and consequently causes a larval–pupal mixed phenotype.

The inhibition of programmed cell death for *E93* RNAi larval epidermis may bring about the failure of ecdysis. As a result, depletion of *E93* at the third- and fourth-instar larval, and the prepupal stages impaired ecdysis in *H. vigintioctopunctata* (Figure 3, Figure 4 and Figure 9). Consistently, the RNAi nymphs in *S. gregaria* fail to molt until death [27].

### 4.3. E93 Specifies Adult Differentiation

It is known that E93 specifies adult differentiation by promoting adult developmental pathways in several hemimetabolans, neometabolans and holometabolans [4]. In *D. melanogaster*, for example, *E93* mutants die as pharate adults [33]. Likewise, the *E93* deletion mutants using CRISPR/Cas9 technology are pupal lethal [4]. A similar phenotype has also been reported in hemimetabolous insects, such as *S. gregaria* [27]. 

Consistently, we found that knockdown of *HvE93* impaired pupal–adult transformation in the present paper (Figure 7). This result indicates that E93 serves as a specifier of adult development in *H. vigintioctopunctata*, in agreement with a central role of E93 established in multiple insect species [5,8,32], using mutants [33], RNAi [26,50] or CRISPR/Cas9 [4]. 

In the present paper, we found that the *HvE93* RNAi *H. vigintioctopunctata* adults had smaller body sizes, wings and legs (Figure 7). These phenotypes resemble those reported in *D. melanogaster*, where the *E93* mutants display widespread defective phenotypes, such as abnormal eye pigmentation, soft adult cuticle, and reduced wing size [4,33,50]. In *T. castaneum*, knockdown of *E93* reduces elytra and hindwings [26]. Our results and those from other insects [4,26,33] reveal that E93 plays a crucial role in the formation of adult appendages during prepupal-pupal-adult transition.

In *D. melanogaster*, E93 promotes Dpp signaling to regulate wing development [50]. Moreover, E93 contributes to the stimulation of *Dll* essential for the development of legs [31,33]. Whether E93 exerts the regulative role of appendage growth through the same signal pathways deserves further experiments to confirm in *H. vigintioctopunctata*.

### 4.4. Mechanism of E93 Controlling Larval Metamorphosis

Our data here revealed that knockdown of *E93* increased the expression levels of *Kr-h1* and decreased the levels of five Halloween genes (Figure 8). Our results indicate that the biosynthesis of 20E is repressed in *E93* RNAi *H. vigintioctopunctata*. Consistently, significant decreases in transcript levels of *HvEcRA*, *HvEcRB1*, *HvUSP*, *HvE74* and *HvFtz-f1* were observed in the ds*E93*-injected prepupae (Figure 8). Our results are consistent with the common notion that increased JH signal inhibits biosynthesis of ecdysone in insects [51,52].

Conversely, the transcription levels of *HvBrC*, *HvE75*, *HvHR3* and *HvHR4* were significantly upregulated in the *E93* RNAi beetles (Figure 8). In agreement with our results, E93 is vital for the proper repression of *BrC* expression in both hemimetabolans and holometabolans [4,9,14,26]. As an early 20E response gene, low level of BrC in the *E93* RNAi beetles cannot stimulate the expression of other 20E response genes, such as *HvE75*, *HvHR3* and *HvHR4* in *H. vigintioctopunctata*. In accordance with our findings, knockdown of *BrC* in another Coleopteran *Dendroctonus armandi* reduces the transcription of E75 and HR3 in the resultant larvae and male and female adults [53]. Similarly, several other 20E response genes, such as *E74A* and *E75A*, are higher in late prepupae in *D. melanogaster E93* mutants, compared with those in controls [4]. More importantly, the functional interaction between E93 and BrC is required for arresting earlier developmental programs associated with BrC function and directing the transition to adulthood [8,32].

Accordingly, rescuing experiments were performed (Figure 9). Dietary supplement with 20E partially restored the decreased larval survivorship and the normal pupa rate. In contrast, a combination of ds*E93*/ds*Kr-h1* injection increased larval mortality but decreased the abnormal pupa rate (Figure 9). These results suggest that disruption of 20E and/or JH signal may be partially responsible for the negative phenotypes in the *E93* RNAi beetles in *H. vigintioctopunctata*.

The suggestion from our rescuing experiment implies that E93 has wide regulative roles for the expression of a lot of genes, except for disruption of 20E and/or JH signal. Our results from rescuing experiment are consistent with a common notion drawn from the documented evidence in *D. melanogaster*, where E93 controls adult differentiation by changing chromatin accessibility in temporally dynamic enhancers of a great number of genes [54,55]. Further research will shed light on this issue.

## 5. Conclusions

In summary, in the present paper, we discovered that the larval instars are fixed in *H. vigintioctopunctata*; E93 acts as a repressor of larval characters and a specifier of adult structures during the larval–pupal–adult transition.

## Data Availability

The data presented in this study are available upon request from the corresponding authors.

## Supplementary Materials

The following supporting information can be downloaded at: https://www.mdpi.com/article/10.3390/biology11111640/s1, Figure S1: Alignment of nucleic acid sequences of *HvE93* isoforms from *Henosepilachna vigintioctopunctata*; Figure S2: Alignment of amino acid residues in ecdysone-induced protein 93F (E93); Figure S3: Phylogenetic analysis (B) of ecdysone-induced protein 93F (E93); Table S1: Primers used in RT-PCR, dsRNA synthesis and qPCR.
