# Supplementary material for: Knockdown of Ecdysone-Induced Protein 93F Causes Abnormal Pupae and Adults in the Eggplant Lady Beetle
Source: Biology (Basel). 2022 Nov 9;11(11):1640. doi: 10.3390/biology11111640 (PMC9687827; doi:10.3390/biology11111640)
Supplement: Supplementary file 1 [file biology-11-01640-s001.zip › biology-1950552-supplementary.pdf]

Supplementary data

Knockdown of *ecdysone-induced protein 93F* causes abnormal pupae and adults in the eggplant lady beetle

Jian-Jian Wu, Feng Chen, Long-Ji Ze, Chen-Hui Shen, Lin Jin, Ahmad Ali Anjum, Guo-Qing Li*

Education Ministry Key Laboratory of Integrated 3Management of Crop Diseases and Pests, College of Plant Protection, Nanjing Agricultural University, Nanjing 210095, China

Table S1. Primers used in RT-PCR, dsRNA synthesis and qPCR

Fragment name	Forward primer	Reverse primer
**RT-PCR**		
HvE93	TGAAGAAGCCCCACGAAA	TTTTCAGCAACACTCGTC
**dsRNA synthesis**		
ds*HvE93*-1	CCCCTTTTGACCCTAACT	CGCTGTCACCGTTCACTT
ds*HvE93*-2	TCCGACAGTCACAGTGAC	ACTTCCCTTACTCAAGTCC
ds*HvKr-h1*-1	AAGTTGCACAGGCACATGA	TTGGTGTAACTTCAGCACG
ds*HvKr-h1*-2	TAAAAGTCACTCGGAAAACG	CGTTCCGTAACAGAGGCA
ds*HvHairy*-1	AGGAGCAACAAGCCTATCA	GCACACTCGCTAAAACCAG
ds*HvHairy*-2	CCCTACTCGTCTGCCCAAT	GGTCTCCAAGGTTTTTCTTCAA
ds*egfp*	AAGTTCAGCGTGTCCG	CACCTTGATGCCGTTC
**qPCR**		
q*HvE93*	GCTCATCCGACCTTTCACCA	TCTTGGGTAGCCGGGTATGA
q*HvE93X1*	AAAAATAGTGGAGAGCAGCCCTT	TCGGCTTCGCTTTGAAAATATGT
q*HvE93X2*	GAGCAGCCCTTGGACTTGAG	CGGCTTCGCTCTAAAATCAGAATT
q*HvKr-h1*	TCCTTCGGTACCATTCACGC	GAGGCGTCAAGATGGGTTCA
q*HvHairy*	AGCAATGGAAACGGTAGTGG	ACTTGATGCAGATGCTGTCG
q*HvEcRA*	GCTCGAAACATGACGTTGCT	TCGCCAATTGATGTGTGGCA
q*HvEcRB1*	GGCCTCCTCACCTCATCAAC	CGAACCGAGTACAAGTCCGT
q*HvUsp*	GGATGCATCAGAGGTGGAGT	AAACTTGGTCACACACTGGC
q*HvE74*	AACCGTCGTTACATTCGCCT	TTGCTGAGGTATGTGGCCTG
q*HvE75*	TAAAAAGTTCCGCGCGCTTC	GTTGGGGTTGCGGAGACATA
q*HvHR3*	CACGACTACCTACGACCCAA	TGAAACGCTCGCTATGATGC
q*HvHR4*	CACGACGTCGGACTGATGAT	GGCACGTCATATACCGCTCA
q*HvBrC*	CCCTGTCCTCCTTCCTCAAG	TGTACTTGCGCCAACTGTTC
q*HvFTZ-F1*	CACATCGACAAGACGCAGAG	ACAGCTTCGAGTTTCATGCC
q*HvSpo*	ATCTGGGAAGCATTGCGACA	TCGGGTTATCCCAGTACGCT
q*HvPhm*	CGGTATTGCAGGGCCACTAA	TGCCAATTTTTCACGAAGCGT
q*HvDib*	GAATCAAGTTGCGTGCAGGT	GCGTGCAATGCAGGATCTAC
q*HvSad*	GTCCTTGCTCGACAATGCTT	TTCGTGAAACGTGCCTGGTA
q*HvShd*	CGCTCTTGTTCTTGGGAGGA	AACTGCGTGTCCCTCAAACT
q*HvRPS18*	CGCAATCAAAGGTGTTGGAAG	GCCTAGGGTTGGCCATAATAG
q*HvRPL13*	AGCATCCTTCGCTCGTTTAG	AGCATCCTTCGCTCGTTTAG

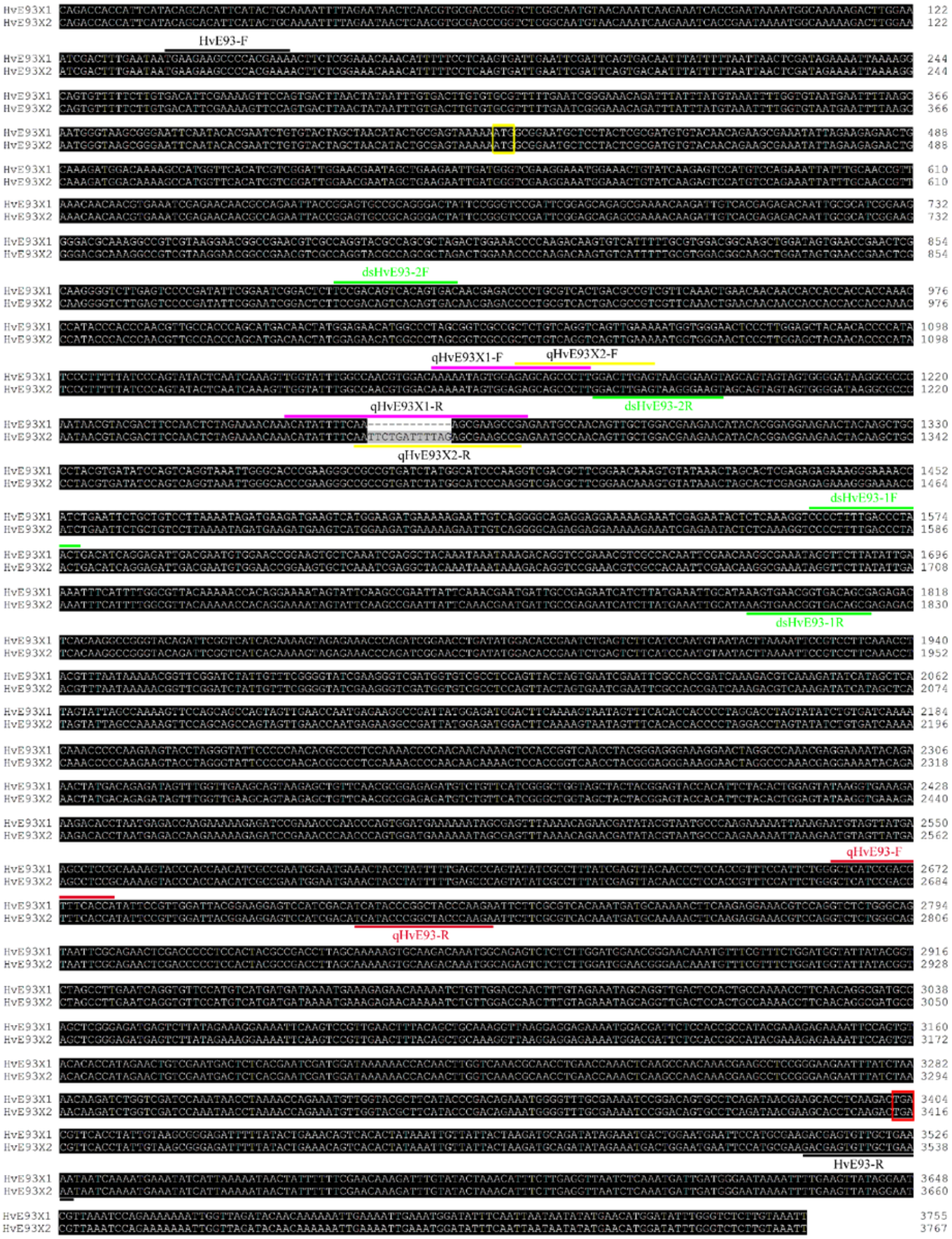

Figure S1. Alignment of nucleic acid sequences of *HvE93* isoforms from *Henosepilachna vigintioctopunctata*. Two *HvE93* isoforms are aligned. The primers for RT-PCR, and the sequences for qRT-PCR are marked; and the sequences of ds*HvE93*-1 and ds*HvE93*-2 are highlighted.

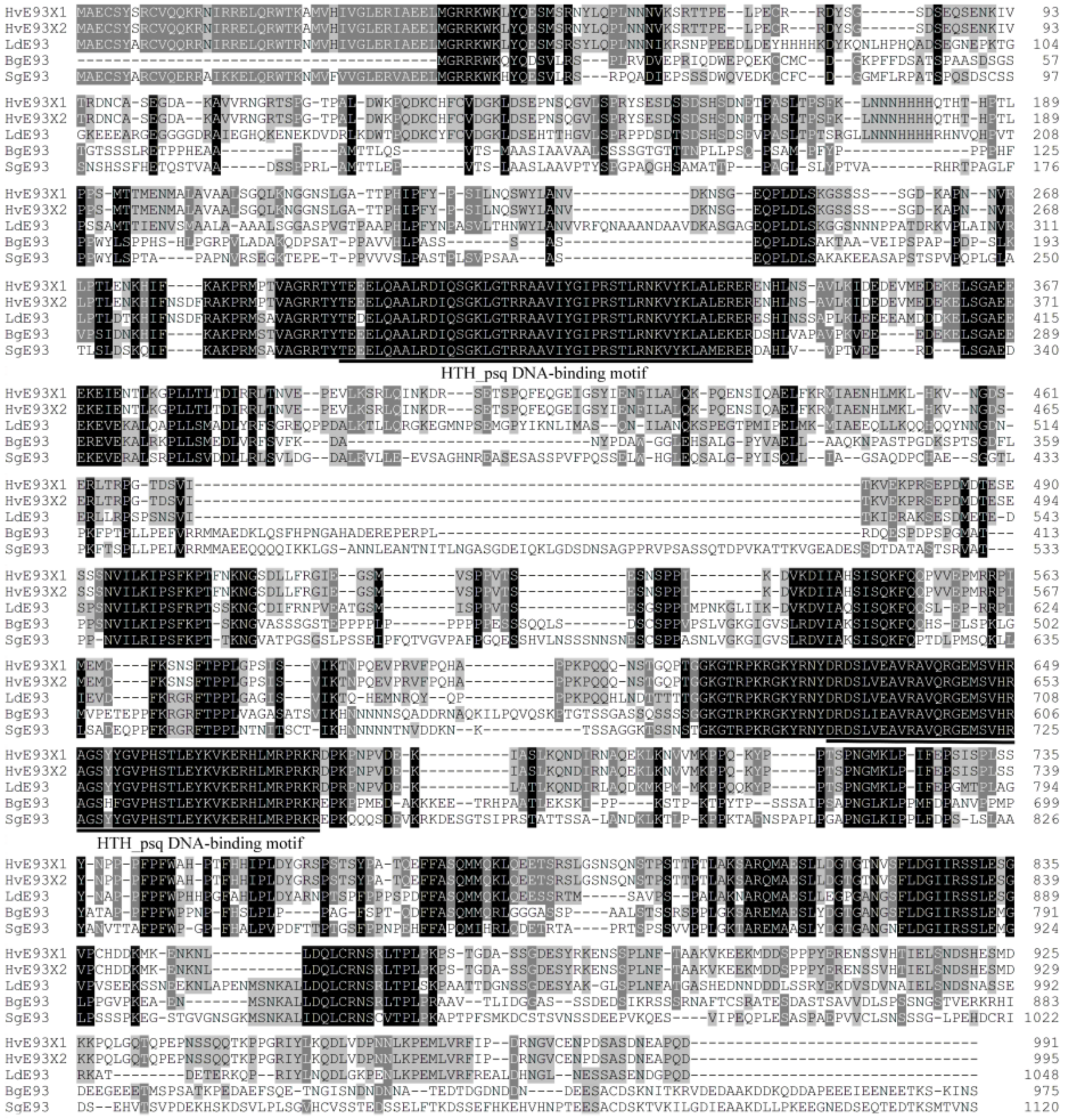

Figure S2. Alignment of amino acid residues in ecdysone-induced protein 93F (E93). E93 proteins are derived from two Coleopteran *H. vigintioctopunctata* (*HvE93X1*, OM001097; *HvE93X2*, OM001098) and *Leptinotarsa decemlineata* (ATI99809.1), an Orthopteran *Schistocerca gregaria* (BBA65756.1), and a Blattarian *Blattella germanica* (CCM97102.1). Increasing background intensity (from light to dark) indicates an increase in sequence similarity. Gaps have been introduced to permit alignment. Two psq-type HTH DNA-blinging motifs are marked with black lines respectively.

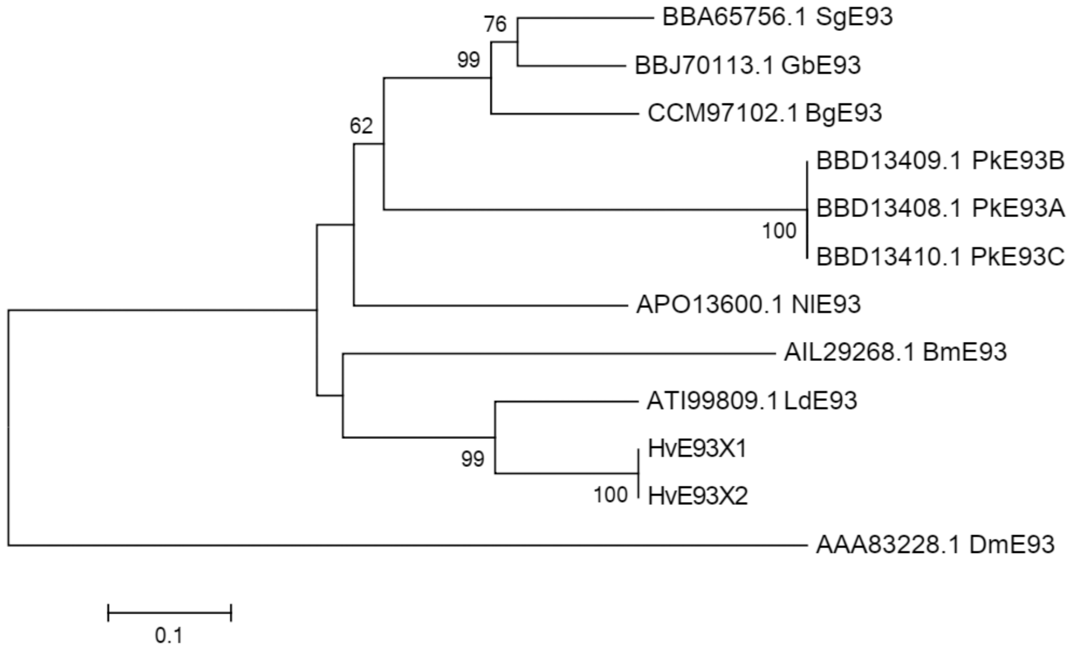

Figure S3. Phylogenetic analysis (B) of ecdysone-induced protein 93F (E93). E93 proteins are derived from two Coleopteran *H. vigintioctopunctata* (*HvE93X1*, OM001097; *HvE93X2*, OM001098) and *Leptinotarsa decemlineata* (ATI99809.1), a Dipteran *Drosophila melanogaster* (AAA83228.1), a Lepidopteran *Bombyx mori* (AIL29268.1), two Hemipteran *Nilaparvata lugens* (APO13600.1) and *Planococcus kraunhiae* (BBD13408.1, BBD13409.1, BBD13410.1), two Orthopteran *Schistocerca gregaria* (BBA65756.1) and *Gryllus bimaculatus* (BBJ70113.1), and a Blattarian *Blattella germanica* (CCM97102.1). The tree is constructed using the neighbour-joining method based on the full-length protein sequence alignments. Bootstrap analyses of 1000 replications are carried out and bootstrap values >50% are shown on the tree.
